# Attention-Enhanced U-Net for Sensor-Efficient High-Density EEG Reconstruction in Wearable Brain Monitoring Systems

**DOI:** 10.1007/s10916-026-02374-5

**Published:** 2026-04-06

**Authors:** Jyun-Rong Zhuang, Pin-Cheng Guo

**Affiliations:** Department of Mechanical Engineering, National Chung Hsing University, 145 Xingda Rd., South Dist, Taichung City, Taiwan 402202 R.O.C.

**Keywords:** Electroencephalography (EEG), Virtual Channel Generation, Sensor Efficiency, U-Net, High-channel-density (HCD), Low-channel-density (LCD)

## Abstract

High-channel-density (HCD) electroencephalography (EEG) enables fine-grained neural sensing but is constrained by high hardware costs, spatial complexity, and limited portability. This study developed a deep learning-based method to reconstruct high-density EEG signals from low-channel-density (LCD) inputs, enabling more practical and affordable brain-monitoring systems. This study introduces VEEG-A-U-Net, a lightweight U-Net architecture enhanced with attention gates and residual learning. The model combined spherical spline interpolation with a learnable correction signal to adaptively model spatial-temporal features. The framework was trained and evaluated on the SEED dataset, using normalized mean square error (NMSE), signal-to-noise ratio (SNR), and Pearson correlation coefficient (PCC) to assess reconstruction performance. Validation was conducted through leave-one-subject-out cross-validation (LOSO-CV) and cross-dataset experiments to examine generalizability. Under the same reconstruction setting (scale factor = 2), VEEG-A-U-Net achieved competitive reconstruction performance compared with state-of-the-art methods, while requiring substantially fewer parameters and computational operations. Cross-dataset evaluations confirmed stable performance across different EEG paradigms. Inference-time analysis showed low computational latency, indicating practical feasibility for deployment in resource-constrained and edge computing environments. A preliminary clinical EEG evaluation was also conducted to explore feasibility in clinical settings.The proposed framework offers an effective and lightweight solution for reconstructing high-density EEG from sparse measurements. These findings may support the development of sensor-efficient and portable EEG systems for practical neuroengineering and brain–computer interface applications.

**Supplementary Information:**

The online version contains supplementary material available at 10.1007/s10916-026-02374-5.

## Introduction

Brain computer interfaces (BCIs) facilitate interaction with external devices by recording and interpreting neural signals, and they have found extensive applications in clinical diagnostics, assistive technologies, and cognitive science research [1, 2]. Among these signals, EEG stands out for its non-invasive nature, relatively low cost, and high temporal resolution. However, the spatial resolution of EEG is heavily dependent on the number of electrodes. Although low-channel-density (LCD) setups offer portability, they typically lack sufficient spatial detail for high-accuracy BCI operations [3, 4]. Conversely, high-channel-density (HCD) configurations provide richer information but are expensive, cumbersome, and time-consuming to set up [5]. This contrast highlights a persistent trade-off in EEG system design. Balancing the trade-off between LCD convenience and HCD precision, therefore, remains a critical research challenge.

To address these issues, some researchers have proposed EEG channel selection techniques that reduce computational loads by focusing on electrodes deemed most relevant for classification or feature extraction [5, 6]. While these methods can improve efficiency, discarding electrodes may remove informative signals and reduce system robustness. An alternative strategy, often termed “virtual EEG channel generation” or “EEG super resolution,” aims to enhance spatial resolution without physically increasing the number of electrodes. By interpolating or learning from available channels, these approaches aim to approximate HCD-level signal detail while preserving the portability and lower cost of LCD systems.

A well-known approach for reconstructing missing or damaged channels is interpolation. Techniques such as nearest neighbor interpolation (NNI), planar spline interpolation (PSI), and spherical spline interpolation (SSI) assign weights to surrounding electrodes based on inverse distance [7]. Soong et al. [7] demonstrated that global interpolation methods, including PSI and SSI, outperform local interpolation (e.g., NNI), but these methods remain sensitive to head-model assumptions and spatial electrode distribution. However, these approaches typically rely on fixed spatial assumptions and uniform weighting schemes, which may inadequately capture sparse and task-relevant neural signals, especially under noisy or ill-conditioned electrode configurations [8]. Extending these methods, Dong et al. [9] proposed a three-concentric- sphere model with improved physiological realism that accounts for scalp conductivity, achieving lower errors and higher correlations than conventional spline algorithms. Liu et al. [10] introduced an attention mechanism to refine channel weighting. However, these interpolation-based strategies may still struggle when reconstructing multiple or edge-located channels, especially under noisy or highly variable conditions.

Deep learning approaches have also emerged for generating EEG channels. Svantesson et al. [11] used a convolutional neural network (CNN) to upsample EEG data in both spatial and temporal dimensions, achieving better reconstruction over interpolation methods. However, the clinical applicability and subject-to-subject generalizability of this model have not been fully established. Li et al. [12] harnessed Wasserstein Generative Adversarial Networks (WGANs) to generate EEG data, demonstrating promising gains in classification accuracy with fewer physical electrodes. However, the number of reconstructed channels remained limited. Tang et al. [13] integrated head-structure and functional connectivity, thereby enhancing reconstruction quality but at the cost of increased computational complexity and runtime. Similarly, Sun et al. [14] employed an informer-based model to create virtual channels, boosting BCI performance but requiring separate models for each channel and substantially increasing computational requirements. More recently, Li et al. [15] proposed an EEG super-resolution framework for emotion recognition using a ResNet-based architecture on the SEED dataset, demonstrating that super-resolved EEG from 32 channels can achieve performance comparable to higher-density recordings. However, their approach focused on feature-level (PSD-based) reconstruction and relied on relatively heavy backbone networks, leaving computational efficiency and time-domain reconstruction largely unexplored.

Despite these advances, existing methods exhibit several recurring limitations. Most do not sufficiently address inter-subject variability: models trained on one group may not generalize well to another without extensive recalibration. Spline-based methods may incur substantial computational cost and remain sensitive to large errors if the scalp geometry or electrode positions deviate from their underlying assumptions. Deep learning solutions often lack clinical validation and may be resource-intensive, especially for high-density reconstructions. Moreover, many of these techniques reconstruct only a limited number of channels, which is insufficient for clinical applications requiring comprehensive spatial coverage. Thus, a gap remains a robust and generalizable solution that can generate high-density EEG signals from low channels while maintaining portability, accuracy, and cost-effectiveness.

The present research addresses this gap by developing a novel deep learning framework for reconstructing HCD EEG data across diverse subjects. The study hypothesizes that an architecture enriched with attention mechanisms (A) will better capture spatiotemporal dependencies, thereby increasing reconstruction accuracy. Although attention-enhanced U-Net architectures have been widely explored in image segmentation and MRI analysis, their application to EEG channel reconstruction has received limited investigation. Unlike imaging tasks that focus on spatial region-of-interest identification, EEG reconstruction requires modeling spatiotemporal dependencies among electrode channels under high noise and inter-subject variability. In this work, attention gates are adapted to regulate feature propagation across skip connections, enabling the network to suppress noise-dominated or less informative channels while emphasizing spatially and temporally significant EEG features. This task-oriented adaptation allows attention mechanisms to serve as an effective, parameter-efficient tool for high-density EEG reconstruction rather than a generic architectural component. The main contributions of this study are as follows:

1. This study proposes VEEG-A-U-Net, a sensor-efficient, task-adapted attention-enhanced U-Net framework for reconstructing HCD EEG signals from LCD inputs. Rather than introducing a new attention formulation, this work is the first to systematically adapt and validate attention gates for high-density EEG channel reconstruction, where attention regulates spatiotemporal feature propagation across electrode channels under noise and inter-subject variability.
2. The study introduces a residual reconstruction strategy that integrates SSI with an attention-guided U-Net framework. By learning to correct interpolation-induced distortions rather than reconstructing signals from scratch, the proposed framework suppresses noise-dominated or less informative features and emphasizes spatially and temporally significant EEG components, thereby improving reconstruction fidelity.
3. The results demonstrate that competitive EEG reconstruction performance can be achieved within a highly lightweight and deployable architecture. Under the same reconstruction difficulty (scale factor = 2), VEEG-A-U-Net achieves reconstruction fidelity comparable to state-of-the-art methods, while requiring substantially fewer parameters and FLOPs. To the best of the authors’ knowledge, this study is the first to demonstrate the practical feasibility of deploying LCD-to-HCD EEG reconstruction on an edge-embedded platform, highlighting its potential for real-world and wearable EEG applications.

From an application standpoint, improving EEG spatial resolution without increasing sensor count could support clinical scenarios that require portable and scalable brain monitoring systems, such as early stroke detection, epilepsy screening, or cognitive fatigue tracking.

## Methods

### Dataset and Preprocessing

This study initially employed an emotion-related EEG dataset to develop and validate our proposed approach. Specifically, the study used the SEED dataset [16] from Shanghai Jiao Tong University, which comprises recordings from 15 participants (7 males and 8 females). Each participant viewed 15 emotional video clips designed to elicit positive, neutral, and negative affective states, while their EEG signals were recorded using a 62-channel device at a sampling rate of 1000 Hz.

To simulate an LCD configuration, the original EEG data were downsampled to 200 Hz, along the electrode dimension a bandpass filter (0–75 Hz) was applied, yielding an LCD dataset that aligns with a commonly used 32-channel EEG setup based on the standard 10–20 system. CB1 and CB2 were excluded from the reconstruction targets, as these mastoid-reference electrodes are located near the ears and are rarely used in standard BCI or EEG analysis pipelines. Next, a sliding window approach was applied to the temporal dimension to segment the time series into 3-second samples. Following segmentation, the sampling rate was maintained at 200 Hz to preserve temporal resolution [17]. By preparing both the LCD-derived samples and their corresponding HCD signals, the dataset was organized for subsequent analysis and model training.

### VEEG-A-U-Net Framework

**Fig. 1:**
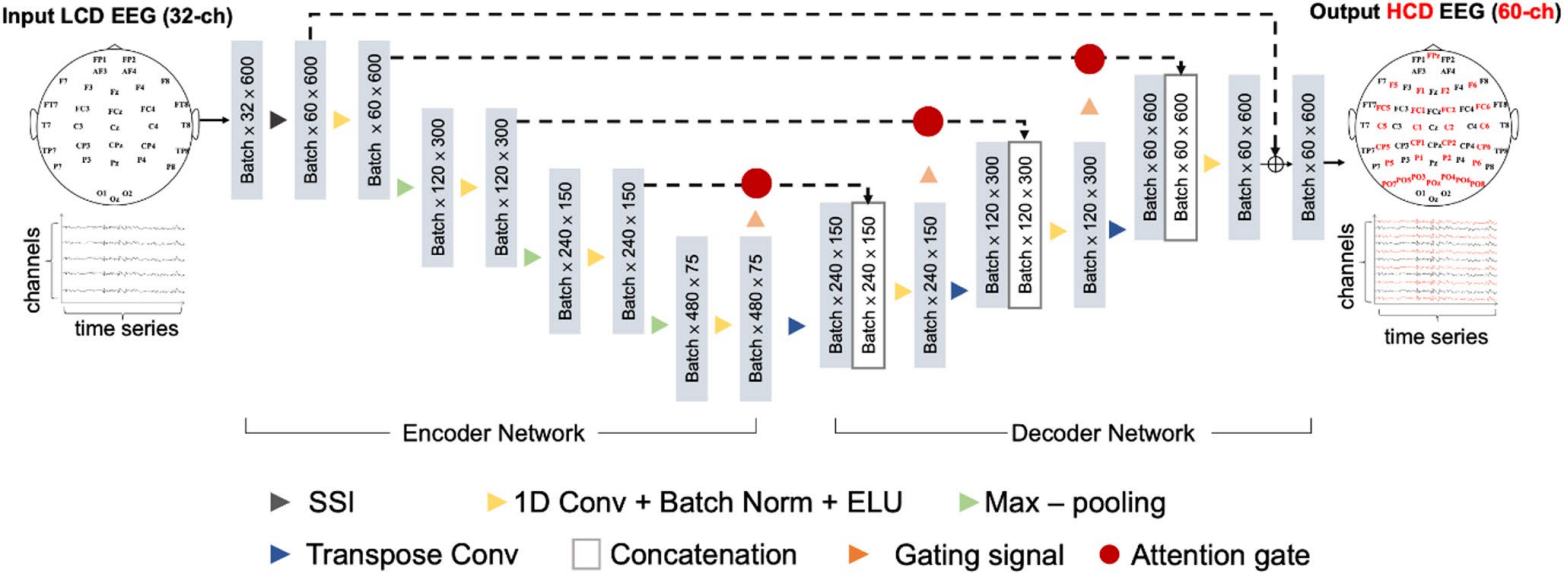
Architecture of the proposed VEEG-A-U-Net framework for virtual EEG channel reconstruction. The model employs a residual learning framework that integrates SSI with an attention-enhanced U-Net. The encoder extracts multi-scale features from interpolated LCD EEG signals, while the decoder reconstructs high-density EEG outputs using skip connections and attention gates. The final output combines the SSI baseline and the learned residual signal. Channels shown in black denote the input LCD channels (32 channels), while channels shown in red denote the reconstructed channels (28 channels)

In both signal and image processing, it is generally assumed that HCD data and their LCD counterparts share the same fundamental (low-frequency) features. However, capturing the residual details that differentiate HCD signals from LCD signals is pivotal in virtual EEG channel reconstruction [18]. To address this challenge, this study proposes VEEG-A-U-Net, a residual-based framework that learns to generate high-fidelity virtual EEG channels, as shown in Fig. 1. The source code and model configurations are publicly available at https://github.com/IASlab526/VEEG-A-U-Net.

#### Spherical spline interpolation (SSI)

As a preliminary step, SSI is applied to estimate missing electrode data, thereby expanding the LCD electrode configuration to match the HCD dimension. This interpolated output serves as an initial approximation of HCD data.

#### Modified Attention U-Net

Next, drawing inspiration from Attention U-Net [19], the study develops a modified U-Net structure to model the residual relationship between LCD and HCD signals. This network consists of two primary stages:

1. Encoder Network.

The encoder progressively extracts features through multiple 1D convolution–batch normalization–ELU (CBE) blocks and max-pooling layers. After each pooling operation, the number of convolution kernels is doubled to capture increasingly abstract and informative patterns.

(2) Decoder Network

The decoder reconstructs high-dimensional signals using transposed convolution (i.e., deconvolution) for upsampling, along with corresponding CBE blocks. Skip connections from the encoder are incorporated at each decoding stage to preserve spatial and temporal cues.

**Fig. 2:**
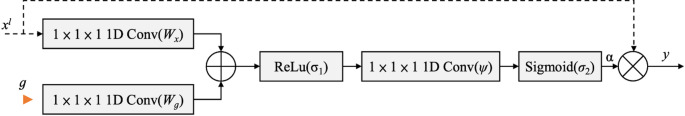
Schematic of the attention gate mechanism illustrating how relevant encoder features are selectively emphasized for improved EEG reconstruction

To enhance feature selectivity, attention gates are inserted before each skip connection (see Fig. 2). Let *x*^*l*^ denote the encoder output at a particular level (*K*×*C*×*T*), and *g* be the corresponding gating signal from the decoder (2*K*×*C*×*T*). Since *g* has larger dimensions, it is first upsampled and convolved together with *x*^*l*^. Their combined output passes through a ReLU function, then a 1 × 1 convolution 𝜓 followed by a Sigmoid to generate the attention matrix 𝛼. The final output *y* is obtained through element-wise multiplication with *x*^*l*^:

1 $$y=AttenGates\left(x^1,g\right)=\alpha\odot x^1$$

2 $$\alpha=Sigmoid\left(\psi(Relu(\;W_x^\ast\;x_1+W_g^\ast g))\right)$$

This process suppresses irrelevant features and highlights significant regions necessary for more accurate reconstruction.

#### Overall Framework and Loss Function

The final reconstruction *Z* from VEEG-A-U-Net is obtained by summing the SSI output and the modified Attention U-Net prediction. The study measures the quality of *Z* relative to the ground-truth HCD signals *X* via the mean squared error (MSE) loss. Adopting and extending the formulation in [20], the model minimizes:

3 $$\begin{array}{c}L=\left\|Z-X\right\|_2^2=\left\|SSI\left(\widetilde X\right)+DE(En(SSI(\widetilde X)))-X\right\|_2^2\\\end{array}$$

where $\widetilde X$ represents the LCD time-series data, *SSI* ($\:\cdot$) denotes the interpolation operation, and *En* and *De* represent the encoder and decoder sub-networks, respectively. The notation $\left\|\cdot\right\|_2^2$ refers to the squared L2-norm, corresponding to the sum of squared differences over all channels and time samples. By optimizing this objective, VEEG-A-U-Net learns latent feature mappings that transform LCD inputs into robust virtual HCD outputs, thereby enabling higher-resolution EEG monitoring without additional hardware costs.

### Performance Evaluation

Experiments were conducted to assess the performance of VEEG-A-U-Net on EEG reconstruction. All models were trained in an offline setting using a workstation equipped with an AMD Ryzen Threadripper PRO 5975WX, 256 GB of RAM, and three Nvidia RTX 4090 GPUs (24GB memory). Training was performed for 50 epochs with a batch size of 64 using the Adam optimizer (learning rate = 0.0035). It should be noted that this hardware configuration was used exclusively for training and does not represent the computational requirements during inference or deployment.

#### Evaluation Metrics

To quantitatively measure the effectiveness of VEEG-A-U-Net in reconstructing HCD signals, the study employs signal-to-noise ratio (SNR) and two additional commonly used metrics, namely normalized mean-squared error (NMSE) and Pearson correlation coefficient (PCC) [21, 22]. SNR gauges the ratio of clean signals to noise, with higher values indicating better signal quality. NMSE provides a standardized measure of the difference between predicted and ground-truth values; unlike conventional MSE, NMSE enables comparisons across datasets with different scales or units. PCC captures the linear relationship between two sets of data (here, the reconstructed signals *Z* and the original *X*) with values ranging from − 1 (perfect negative correlation) to + 1 (perfect positive correlation).

#### Leave-One-Subject-Out Validation

Because EEG data in biomedical research are often collected from multiple participants, leave-one-subject-out cross-validation (LOSO-CV) is adopted in this study to examine the model’s generalizability to previously unseen subjects. Under LOSO-CV, data from a single participant serves as the validation set, while the remaining participants’ data constitute the training set. In this study, the training set is further split into 80% for training and 20% for testing, and the designated validation set is used to assess model performance using the evaluation metrics. This approach evaluates whether the trained model extends beyond the subjects involved in its training process.

## Results

### EEG reconstruction results

**Fig. 3:**
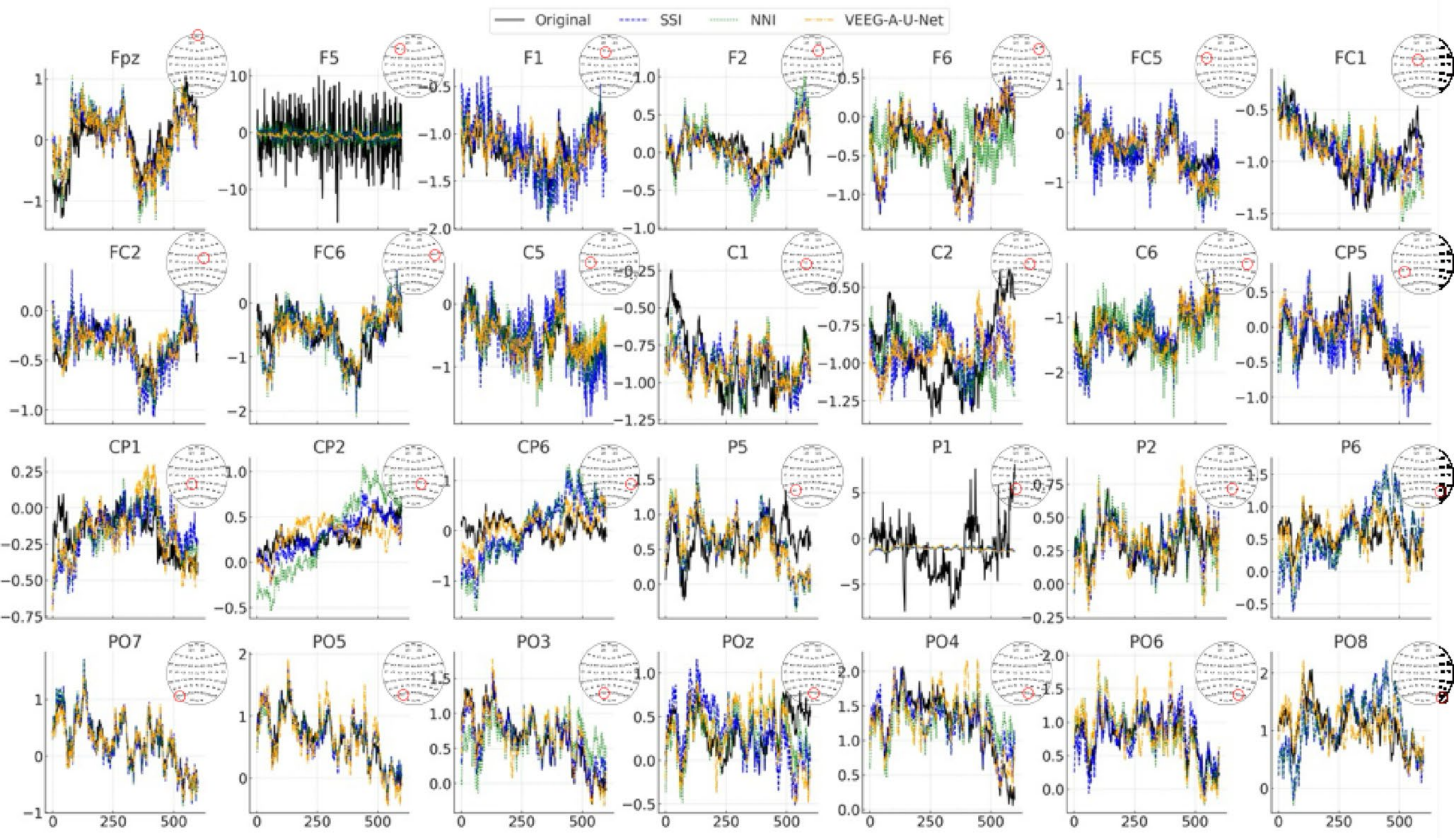
Comparison of reconstructed EEG time-series signals for Original EEG (black line), SSI (blue dashed line), NNI (green dotted line), and VEEG-A-U-Net (yellow dashed line) across 28 reconstructed channels, highlighting waveform fidelity and noise reduction characteristics

To gain a clearer understanding of the effectiveness of the proposed model in reconstructing virtual EEG channels, the study first evaluated its performance in the time domain using test data from the SEED dataset. Specifically, the time-series waveforms of the original EEG signals were compared with those reconstructed using three methods of SSI, NNI, and the proposed VEEG-A-U-Net across 28 channels that differed between LCD and HCD configurations. As shown in Fig. 3, the black line represents the original HCD EEG signal, while the colored lines represent the reconstructed outputs: SSI (blue dashed line), NNI (green dotted line), and VEEG-A-U-Net (yellow dashed line).

Overall, compared to traditional methods, VEEG-A-U-Net produced smoother reconstructions that more closely followed the temporal dynamics of the original signals. Although the model preserved the overall trends, it slightly underestimated peak amplitudes, reflecting a bias toward smoother estimation. In contrast, SSI and NNI exhibited visible waveform distortions. NNI frequently produced sharp fluctuations due to its reliance on localized interpolation, while SSI showed considerable amplitude attenuation, particularly in channels where spatial inference was difficult due to sparsely distributed neighboring electrodes. Furthermore, NNI often introduced phase misalignments, resulting in temporally shifted reconstructions.

To quantify these observations, PCC, NMSE, and SNR were computed for each method compared to the original signals, as shown in Table 1. VEEG-A-U-Net achieved the best performance across all metrics, with the highest PCC and lowest NMSE, indicating superior accuracy and signal fidelity. In contrast, SSI and NNI exhibited weaker correlations due to phase distortion and noise amplification. The improvements in SNR further demonstrated that VEEG-A-U-Net produced cleaner, less noisy reconstructions than the baseline methods.

**Table 1:** Comparison of PCC, NMSE, and SNR performance across different reconstruction methods (SSI, NNI, VEEG-A-U-Net) for all reconstructed channels

Method	PCC	NMSE	SNR (dB)
SSI	0.585 ± 0.000	0.684 ± 0.000	1.647 ± 0.000
NNI	0.698 ± 0.092	0.512 ± 0.098	4.760 ± 1.440
VEEG-A-U-Net	0.801 ± 0.121	0.309 ± 0.140	5.925 ± 1.713

The study further analyzed the region-wise reconstruction quality of VEEG-A-U-Net. In the frontal region, except for F5, the model’s outputs closely matched the original waveforms, again showing a tendency toward slight peak attenuation. In the central region (e.g., C1, C2, CP1, CP2), the model accurately reconstructed stable segments, but it diverged considerably during rapid fluctuations. This pattern suggests that the model interpolated signals in both anterior-posterior (e.g., FC to CP) and lateral (left-right hemispheric) directions. However, reconstruction accuracy remained limited for centrally located channels with larger spatial distances from surrounding electrodes (e.g., near C1 and CP2).

In the parietal and occipital regions (e.g., P1, P6, POz, PO4), the reconstructed signals exhibited greater variation, indicating that reconstruction performance in posterior regions was highly dependent on the spatial proximity of the known input channels. Despite access to nearby electrodes (e.g., P3, P4, O1, O2), the model still produced reconstruction errors likely due to edge-related extrapolation effects at the periphery of the electrode montage. Notably, all three methods showed elevated reconstruction errors at channels F5 and P1. A detailed inspection revealed that this behavior resulted from large-amplitude transient activity in the original signal, which posed a fundamental challenge for both interpolation-based and learning-based reconstruction approaches. Therefore, when applying data-driven reconstruction, selecting high-quality input channels is essential to avoid propagating noise into the reconstructed output.

To further quantify regional differences, region-wise average PCC scores were computed (Table 2). Overall, VEEG-A-U-Net achieved comparable or improved reconstruction fidelity relative to SSI and NNI across most regions, with the clearest gains in the frontal region and competitive performance in the parietal region. In the central region, SSI yielded slightly higher PCC, while in the occipital region, VEEG-A-U-Net performed comparably to SSI and outperformed NNI. The parietal region consistently showed the lowest PCC scores across all methods, indicating that accurate reconstruction in this area was more challenging, potentially due to reduced spatial redundancy and less informative neighborhood channels. Taken together, regions with denser electrode coverage yielded more accurate reconstructions, while peripheral and centrally isolated channels showed greater errors. These findings highlight the potential value of incorporating region-specific enhancements, such as adaptive spatial weighting or attention mechanisms, to further improve model generalizability across the scalp.

**Table 2:** Region-wise PCC analysis of time-series reconstruction fidelity across frontal, central, parietal, and occipital regions (28 channels)

Brain Region	SSI (PCC)	NNI (PCC)	VEEG-A-U-Net (PCC)
Frontal (FPz, F5, F1, F2, F6)	0.619 ± 0.370	0.480 ± 0.442	0.662 ± 0.317
Central (FC5, FC1, FC2, FC6, C5, C1, C2, C6)	0.639 ± 0.219	0.589 ± 0.312	0.609 ± 0.245
Parietal (CP5, CP1, CP2, CP6, P5, P1, P2, P6)	0.407 ± 0.347	0.439 ± 0.359	0.446 ± 0.341
Occipital (PO7, PO5, PO3, POz, PO4, PO6, PO8)	0.761 ± 0.279	0.676 ± 0.241	0.761 ± 0.259

**Fig. 4:**
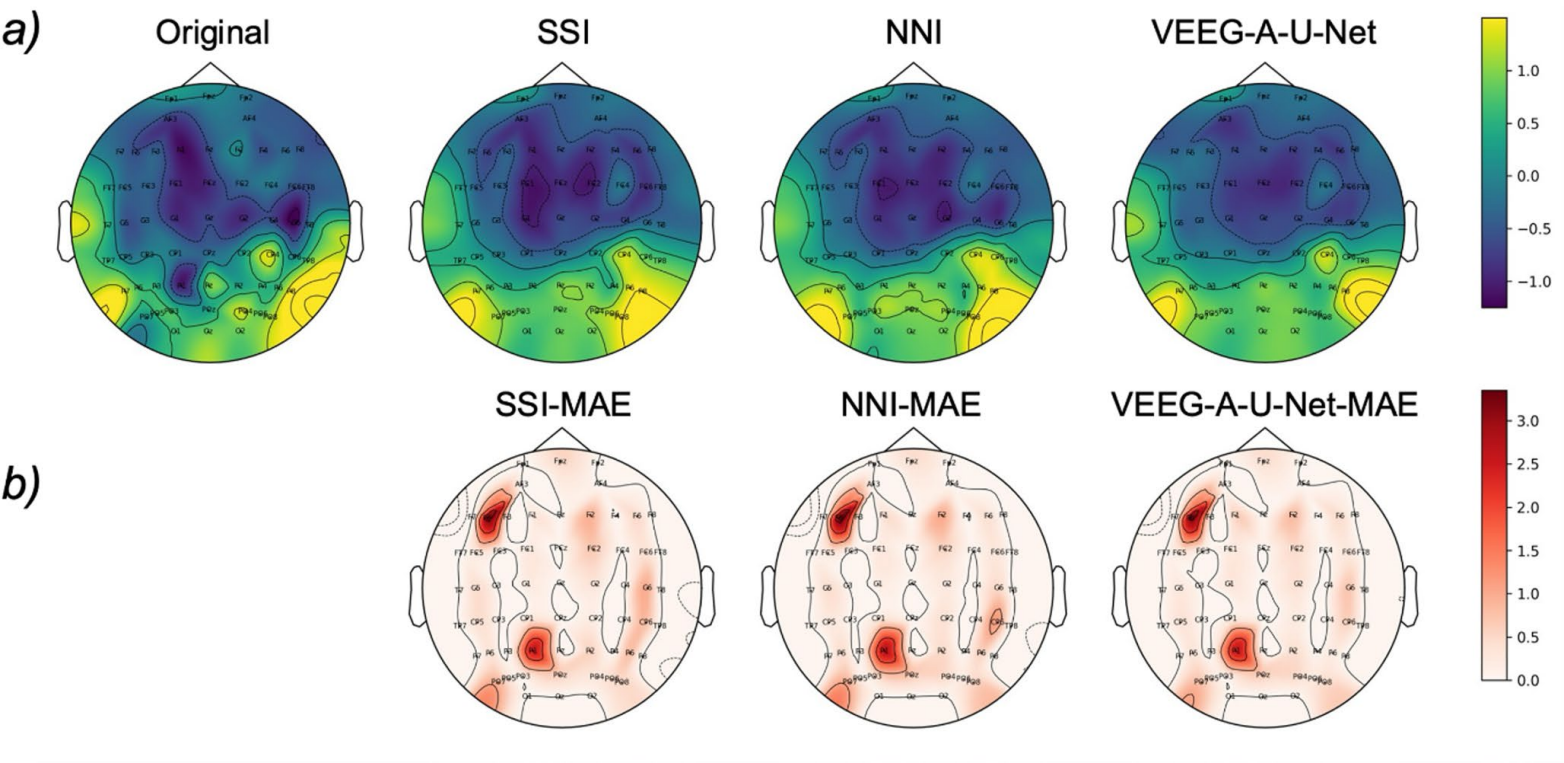
Topographical distribution of the EEG signal and reconstruction errors. (**a**) Time-averaged scalp distributions of the original HCD EEG signals and those reconstructed using SSI, NNI, and VEEG-A-U-Net, and (**b**) channel-wise MAE across all temporal samples for each reconstruction method

Spatial performance was further visualized using topographic maps (Fig. 4a), which show the time-averaged signal distributions of the original EEG, SSI, NNI, and VEEG-A-U-Net. Additionally, the channel-wise mean absolute error (MAE) was computed and visualized across all temporal samples for each channel (Fig. 4b) to assess spatial reconstruction fidelity. VEEG-A-U-Net consistently achieved lower MAE across most scalp regions than traditional methods. Specifically, the proposed method achieved the lowest overall error of 0.262 ± 0.253, outperforming both SSI (0.320 ± 0.276) and NNI (0.305 ± 0.296). Together, both visual and quantitative analyses support the effectiveness of VEEG-A-U-Net in reconstructing high-density EEG signals with improved spatial and temporal fidelity.

### Model Evaluation

To determine the impact of attention gates on the proposed framework, VEEG-U-Net and VEEG-A-U-Net (ablation study) were compared under a leave-one-subject-out cross-validation (LOSO-CV) scheme on the SEED dataset. Table 3 presents the results for the three key metrics (NMSE, SNR, and PCC) across 15 subjects. VEEG-U-Net achieved average NMSE, SNR, and PCC scores of 0.384, 4.376 dB, and 0.782, respectively. By adding attention gates, VEEG-A-U-Net improved performance on all metrics, yielding values of 0.309, 5.925 dB, and 0.801. These correspond to improvements of 19.539% in NMSE, 1.549 dB in SNR, and 2.383% in PCC. Notably, NMSE and PCC also exhibited lower standard deviations, indicating the model’s ability to adapt dynamically to differing data characteristics.

**Table 3:** Performance comparison between VEEG-U-Net and VEEG-A-U-Net under LOSO-CV on the SEED dataset, highlighting improvements introduced by attention gates

	NMSE		SNR		PCC	
	U-Net	A-U-Net	U-Net	A-U-Net	U-Net	A-U-Net
S1	0.808	0.646	0.791	2.574	0.444	0.461
S2	0.277	0.249	5.461	6.345	0.839	0.859
S3	0.249	0.183	5.416	7.628	0.848	0.895
S4	0.254	0.201	5.716	7.327	0.857	0.889
S5	0.222	0.244	5.281	6.431	0.838	0.860
S6	0.551	0.346	0.708	5.814	0.708	0.736
S7	0.179	0.375	5.372	4.351	0.843	0.805
S8	0.283	0.285	5.668	5.623	0.853	0.834
S9	0.266	0.194	5.259	7.413	0.842	0.889
S10	0.682	0.387	3.297	4.675	0.727	0.736
S11	0.247	0.135	5.595	9.033	0.852	0.927
S12	0.357	0.249	4.817	6.365	0.823	0.846
S13	0.372	0.273	4.692	6.431	0.793	0.826
S14	0.377	0.270	5.297	6.236	0.836	0.848
S15	0.629	0.598	2.266	2.622	0.629	0.598
Ave.	**0.384**	**0.309**	**4.376**	**5.925**	**0.782**	**0.801**

Despite VEEG-A-U-Net’s robust gains in signal quality, a few outlier subjects still demonstrated suboptimal results due to inherently low SNR in the original data. In such cases, the potential for improvement was limited by poor input quality. This observation suggests that future EEG reconstruction research could incorporate enhanced noise-reduction algorithms during preprocessing or explore improvements in electrode hardware design. Overall, incorporating attention gates allowed the model to focus more effectively on relevant features while reducing redundant information.

### Comparison with Existing Methods

The study further compared the proposed VEEG-A-U-Net with existing state-of-the-art EEG reconstruction methods (EEGSR-GAN [6], Deep-CNN [21], Deep-EEGSR [13] and, MASER [23], ESTformer [24] and ESTformer+Deep-CNN [24]) using the SEED dataset. Table 4 summarizes the performance of several models across NMSE, SNR, and PCC. Among the compared methods, VEEG-A-U-Net consistently showed comparable performance across NMSE, SNR, and PCC under the LOSO evaluation protocol. In comparison, Deep-EEGSR [13] achieved an NMSE of 0.413, an SNR of 3.843 dB, and a PCC of 0.762. MASER [25] and ESTformer-based approaches achieved competitive PCC values (0.877 for MASER and 0.852 for ESTformer+Deep-CNN), while ESTformer+Deep-CNN further increased SNR to 5.634 dB. Compared with the strongest performance in Table 4, ESTformer+Deep-CNN [23], the proposed method showed a slightly higher NMSE and lower PCC. Overall, the two methods exhibited comparable reconstruction fidelity at a scale factor of 2, with VEEG-A-U-Net providing a clearer advantage in noise-robust reconstruction as reflected in SNR.

**Table 4:** Performance comparison (scale factor of 2) between VEEG-A-U-Net and state-of-the-art EEG reconstruction methods (EEGSR-GAN, Deep-CNN Deep-EEGSR, MASER, ESTformer, and ESTformer+Deep-CNN) on the SEED benchmark dataset

Model	NMSE	SNR	PCC	FLOPs (G)	Params (M)
EEGSR-GAN [6]	0.514	2.892	0.693	3332.2	16.5
DEEP-CNN[18]	0.485	3.143	0.713	939.5	2.2
DEEP-EEGSR[12]	0.413	3.843	0.762	0.62^a^	0.21
MASER[25]	0.274	N/A^b^	0.877	N/A^b^	N/A^b^
ESTformer [22]	0.315	5.027	0.827	1.5	33.7
ESTformer+Deep-CNN [22]	0.274	5.634	0.852	941	35.9
VEEG-A-U-Net	0.309	5.925	0.801	0.164	0.016
a The FLOPs of Deep-EEGSR were estimated based on the network architecture reported in the original paper (composed of convolutional and dense layers), following the FLOPs calculation definitions and conventions described in [26]. When implementation details such as stride or padding were not explicitly specified, commonly adopted conservative assumptions (e.g., stride = 1 with temporal length preserved) were applied to ensure a consistent and reproducible estimation procedure. b N/A: For MASER, the original paper does not provide publicly available source code, and key architectural details (e.g., layer dimensions and hyperparameter configurations) are insufficiently disclosed. As a result, we were unable to compute its parameter count and FLOPs under a consistent and reproducible protocol; therefore, it is labeled as N/A in the table.					

Beyond performance, a key advantage of VEEG-A-U-Net is its architectural simplicity. As shown in Table 4, VEEG-A-U-Net demonstrated a clear efficiency advantage over existing methods. Under the same reconstruction difficulty (scale factor of 2), it requires only 0.164 GFLOPs and 0.016 M parameters—substantially fewer than most competing approaches and lighter than other existing baselines. Despite this markedly reduced model complexity and the absence of additional sophisticated modules, VEEG-A-U-Net achieved reconstruction performance comparable to that of current state-of-the-art methods. These results demonstrated that, under equivalent reconstruction settings, integrating attention gates into a U-Net architecture was sufficient to enable competitive EEG reconstruction within a lightweight framework. Moreover, the proposed framework was inherently flexible by increasing network depth and width within the same design paradigm. VEEG-A-U-Net retained the potential to further enhance performance, rivaling or even surpassing models targeting larger scale factors (e.g., 4×, 8×, or 16×). This scalability underscores the practical value of the proposed approach for applications that demand higher reconstruction fidelity while preserving computational efficiency.

### Cross-Dataset Validation

To further evaluate model performance and generalizability beyond the SEED benchmark, the proposed method was applied to additional datasets. Following the same methodological steps, each new dataset underwent preprocessing before training and evaluation. Additional adjustments were implemented to account for variations in EEG channel counts across these datasets, ensuring consistent output dimensions and enabling fair comparisons.

For rigor and accuracy, this stage used the best-performing model identified in earlier experiments, trained via a mixed-subject strategy. In other words, data from all subjects in a given dataset were combined into a single training set, thereby exposing the model to a diverse range of data characteristics and enhancing its overall adaptability. This design simulates real-world scenarios where data may originate from heterogeneous sources, thereby testing the model’s cross-dataset applicability.

The primary objective was to conduct an initial assessment of the best-performing model’s stability and suitability in multiple datasets, as well as its behavior under differing contexts and data characteristics.

#### Other datasets

Below is an overview of the additional datasets:

1. **Motor Imagery (MI) Dataset** [26].

This dataset comprises EEG recordings from 109 volunteers, acquired with a 64-channel setup at a sampling rate of 160 Hz [26]. Each participant performed multiple motor imagery (MI) tasks—either physically or mentally executing hand or foot movements. Data were resampled at 200 Hz. An 8-channel (FP1, FP2, F3, F4, Cz, P3, P4, Oz) configuration was adopted as the LCD input, while the 32-channel configuration (as shown in Fig. 1-LCD) served as the reconstruction target to generate the remaining channels.

(2) **Mental Fatigue (MF) Dataset** [27].

Collected from a lab at National Chung Hsing University, this dataset contains EEG signals from 10 healthy participants [27]. Recordings were made via a 32-channel EEG device operating at 500 Hz. Each participant underwent a mental fatigue (MF) induction experiment. Because the EEG system supported only 32 channels (identical to Fig. 1-LCD), an 8-channel configuration was adopted to generate the remaining channels.

(3) **Clinical EEG Evaluation (Pilot Study)**.

To evaluate the robustness of the proposed method under realistic clinical conditions, a preliminary clinical EEG evaluation was conducted using recordings collected at Kaohsiung Medical University Chung-Ho Memorial Hospital. The pilot study involved a 77-year-old male patient with a right-sided ischemic stroke. EEG data were acquired using a 32-channel system at a sampling rate of 500 Hz (St. EEG VEGA, Artisebio). During the experiment, the participant performed MI tasks involving imagined up–down and left–right movements of both hands. Each trial consisted of an initial 30-second resting period, followed by motion videos presented on a screen to guide MI execution (80 s). Each movement pattern was repeated five times, yielding a total of 40 EEG recordings. All experiments were conducted under the supervision of experienced clinical physicians. This pilot demonstration was conducted for methodological validation only and did not constitute a clinical trial; clinical trial number: not applicable.

Following the same preprocessing and reconstruction pipeline as in previous experiments, an 8-channel configuration was adopted as the LCD input to reconstruct the remaining channels in an 8-to-32 setting. Owing to the limited number of clinical recordings, this evaluation was conducted as a pilot feasibility study to examine whether the proposed framework could retain meaningful reconstruction capability in a real clinical scenario.

#### Cross-Dataset and Clinical Reconstruction Performance

Table 5 summarizes the cross-dataset reconstruction capabilities of VEEG-A-U-Net. In the MI dataset, the study compared two different LCD configurations, 8 and 32 channels, both reconstructed to 60 HCD channels. The results showed that, for the 8-channel LCD configuration, the model achieved an NMSE of 0.354, an SNR of 4.989, and a PCC of 0.785, whereas the 32-channel setup yielded an NMSE of 0.214, an SNR of 9.597, and a PCC of 0.927. These findings indicate a substantial performance boost with increased original channel count. In the MF dataset, reconstructing from an 8-channel LCD to a 32-channel HCD configuration yielded an NMSE of 0.355, an SNR of 5.917, and a PCC of 0.839. These findings suggest that the proposed model adapted to and reconstructed diverse EEG data, demonstrating generalization to datasets with varying characteristics. Moreover, the clear trend across both datasets was that using a higher initial number of physical electrodes reduced NMSE, increased SNR, and increased correlation (PCC). This observation provides practical guidance for settings where budgets or equipment availability limit channel counts: maximizing the initial electrode configuration, in tandem with the proposed framework, could yield more accurate virtual HCD reconstructions under resource constraints.

**Table 5:** Cross-dataset and clinical reconstruction performance of VEEG-A-U-Net, evaluating the generalization across Motor Imagery, Mental Fatigue datasets, and preliminary clinical EEG recordings

Dataset	LCD (initial set)	HCD (reconstruction)	NMSE	SNR	PCC
MI[23]	8	60	0.354	4.989	0.785
	32	60	0.214	9.597	0.927
MF[24]	8	32	0.355	5.915	0.839
Clinical EEG (pilot)	8	32	0.496	3.173	0.709

In addition to research-grade datasets, preliminary results obtained from clinical EEG recordings are also summarized in Table 5. As expected, reconstruction performance was reduced under clinical conditions; however, the model remained capable of capturing meaningful signal correlations, supporting its feasibility in real-world clinical environments. Although limited in scale, this pilot clinical evaluation demonstrates the potential applicability of the proposed framework under realistic clinical conditions and, to the best of the authors’ knowledge, is one of the first attempts to investigate low-density to high-density EEG reconstruction in a clinical setting.

## Discussions

This study investigated whether a deep learning–based virtual EEG channel generation framework could effectively balance the trade-off between LCD portability and HCD signal fidelity. The primary hypothesis was that incorporating attention mechanisms would enable the model to better capture critical EEG features, thereby improving reconstruction accuracy without requiring additional hardware. Results across multiple datasets partially confirm this hypothesis: VEEG-A-U-Net consistently outperformed traditional interpolation methods (NNI, SSI) and achieved comparable or improved performance relative to several state-of-the-art deep learning approaches [6, 13, 21, 23, 24], with notable gains observed in NMSE, SNR, and PCC. These findings support the effectiveness of integrating attention gates for emphasizing significant EEG components critical for accurate reconstruction.

### Interpretation of Attention Weight Distributions

It is important to note that the attention gates employed in VEEG-A-U-Net were not introduced as a novel architectural invention, but rather as a task-adaptive mechanism tailored for EEG channel reconstruction. Specifically, the attention modules were designed to selectively regulate the flow of encoder features derived from SSI-based interpolated signals before they were propagated through skip connections. By modulating the contribution of spatially neighboring electrodes, the attention gates suppressed irrelevant or noise-dominated features while preserving relevant spatial information critical for accurate reconstruction. To further examine the behavior of the attention mechanism, the study provides qualitative visualizations of attention weight distributions at the individual-subject level in the Supplementary Materials (Figs. S1-S16). These results indicate that the model consistently assigns higher attention weights to spatially informative channels surrounding the reconstruction targets, supporting the effectiveness of attention-guided feature selection in improving reconstruction fidelity.

**Fig. 5:**
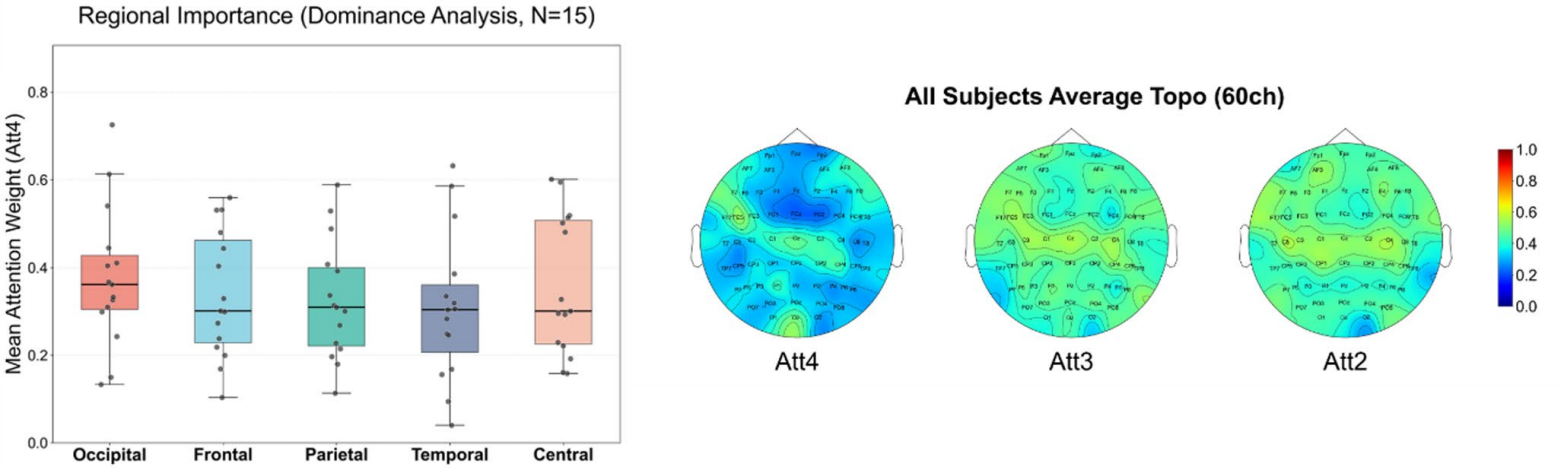
Visualization of attention weight distributions in the proposed VEEG-A-U-Net. Right: Scalp topographies of attention weights averaged across all 15 subjects, extracted from the three decoder attention gates (Att4, Att3, and Att2), corresponding to progressively earlier decoding stages. Left: Region-wise distributions of mean attention weights derived from Att4 across all subjects, showing consistently higher attention in the occipital region, which indicates task-adaptive spatial emphasis during EEG reconstruction

While the overall performance gains are evident, the underlying mechanisms responsible for these improvements warrant further examination. One plausible explanation is that the attention gates dynamically reweight encoder features, enabling the network to emphasize spatially and temporally informative signals while suppressing noise-dominated components. This behavior is consistent with prior EEG studies showing that sparse, task-relevant feature selection is critical for robust neural signal analysis and classification [25]. In addition, the residual learning strategy may help correct distortions introduced by spline-based interpolation. To further substantiate this hypothesis, Fig. 5 presents the attention weight distributions averaged across all 15 subjects to illustrate the global spatial allocation of the attention gates. The results reveal a non-uniform attention pattern rather than homogeneous channel weighting, indicating that the model emphasized specific spatial regions. Notably, the occipital region exhibited the highest relative attention importance in approximately 53.3% of the subjects. This tendency is consistent with the visual-stimulus-driven nature of the SEED dataset, suggesting that the attention mechanism adapts to task-relevant neurophysiological characteristics rather than performing indiscriminate feature scaling.

### Model Simplicity, Computational Efficiency, and Deployment Feasibility

Importantly, the proposed method achieves these improvements using a relatively simple architecture, especially when compared to more complex generative models such as EEGSR-GAN [6] or deep feature extractors like Deep-CNN [21] and Deep-EEGSR [13]. Unlike these prior methods, which typically rely on adversarial training, multi-branch encoders, or spatiotemporal fusion blocks, VEEG-A-U-Net is built upon a straightforward U-Net backbone enhanced with attention gates. This streamlined design offers better performance while maintaining architectural transparency and ease of implementation. The model’s simplicity also facilitates adaptation to other EEG domains without extensive tuning or computational overhead, making it a practical alternative to more elaborate and resource-intensive frameworks.

From a deployment perspective, it is important to distinguish between offline training and online inference. While multiple high-end GPUs were used during training to accelerate the optimization process, this choice did not reflect the computational requirements during deployment. In practical use, only inference is required, and the proposed VEEG-A-U-Net exhibits low computational complexity and latency. Specifically, for a 1 × 60 × 600 input segment (corresponding to a 3-second EEG window at 200 Hz), the model contains only 0.01624 M parameters and requires 0.164 GFLOPs under FP32 precision [28]. Empirical inference-time evaluation further demonstrated that the model achieved an average latency of 13.936 ms on a CPU and 6.007 ms on a GPU, corresponding to real-time factors (RTF) of approximately 215× and 499× (RTF= 3 s/latency per sample), respectively. These results indicate that the proposed model substantially exceeded real-time processing requirements, even without GPU acceleration.

**Fig. 6:**
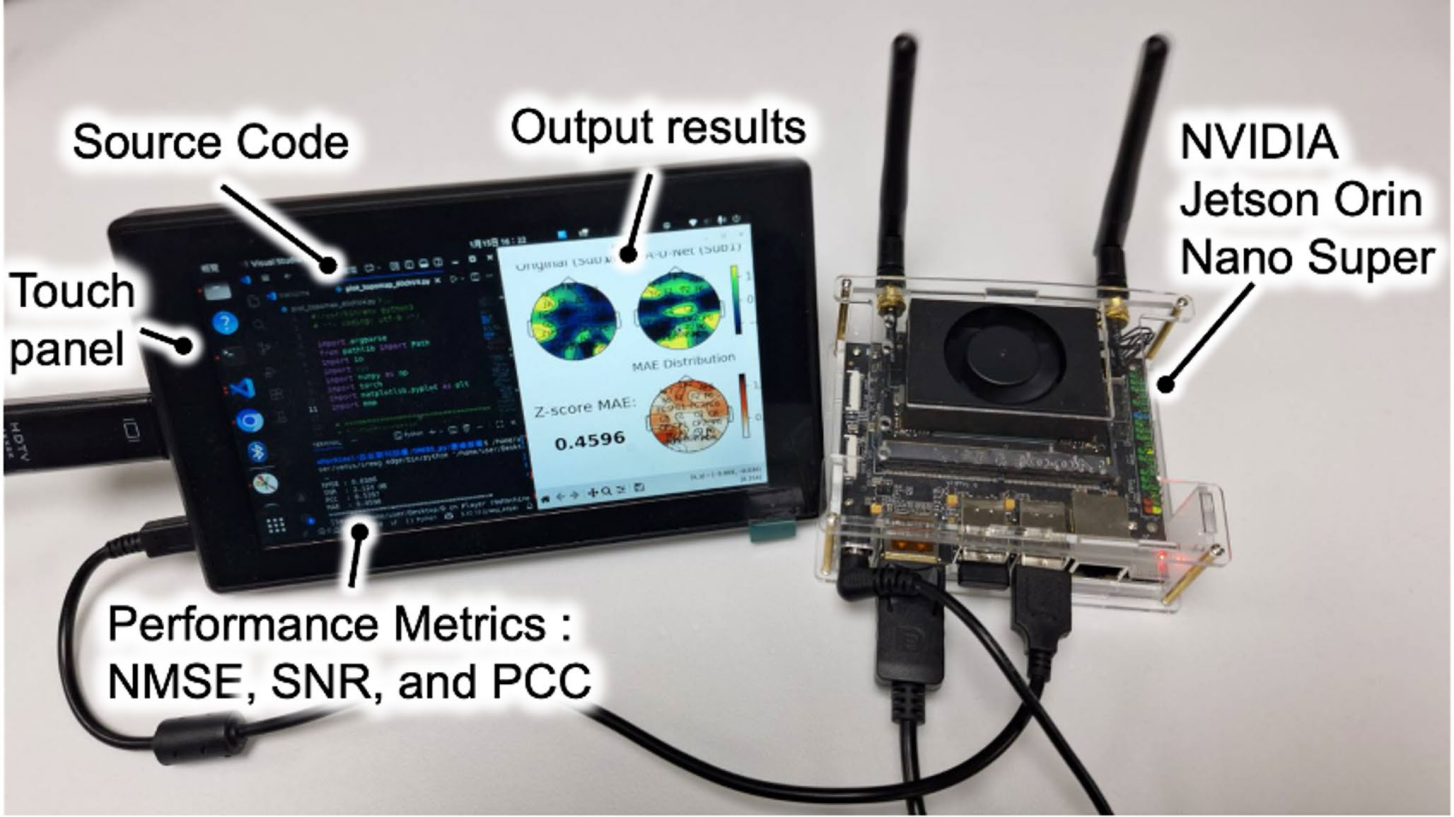
Deployment of the proposed VEEG-A-U-Net on an NVIDIA Jetson Orin Nano Super embedded platform

To further validate edge deployment feasibility, the trained model was deployed and executed on an NVIDIA Jetson Orin Nano Super embedded platform, confirming its suitability for wearable and edge-based EEG applications, as shown in Fig. 6. To the best of the authors’ knowledge, this study is the first to successfully demonstrate low-density to high-density EEG reconstruction deployed and executed on an edge embedded device.

### Limitation

Despite the encouraging results, several limitations were identified. Although VEEG-A-U-Net is lightweight relative to existing EEG reconstruction models, scalability remains an important consideration. Extending the framework to higher-density EEG configurations or more stringent real-time scenarios may require additional optimization strategies, such as model compression or efficient attention variants. Interpretability also remains a challenge: while attention gates provide partial transparency by highlighting spatially informative regions, the internal representations of deep networks are not yet fully intuitive for clinical use. Moreover, the current evaluations were primarily conducted offline using standardized preprocessing, and the robustness of the model under artifact-heavy or highly noisy clinical EEG conditions warrants further investigation.

This study primarily focused on a scale factor of 2, which represents a widely adopted and practically relevant setting for wearable EEG systems and enables fair comparison with existing methods. Under this comparable scale factor, VEEG-A-U-Net demonstrated that a highly lightweight architecture achieved reconstruction performance comparable to more complex models while substantially reducing computational cost. When the scale factor increased (e.g., 4 or 8), the performance gap between lightweight and high-capacity models became more pronounced, as larger-scale reconstruction typically benefits from deeper and wider networks. In such scenarios, the current lightweight configuration is not optimized to compete directly with heavily parameterized models. Nevertheless, this observation does not undermine the potential of the proposed framework. The core design of VEEG-A-U-Net—including SSI-based initialization, residual learning, and attention-guided feature modulation—remains applicable to higher scale factors. By appropriately scaling network depth and width within the same architectural framework, the model is expected to achieve competitive performance at larger reconstruction ratios.

Future research are therefore expected to explore systematic scaling strategies to extend the proposed approach to higher-density reconstruction tasks while maintaining a favorable balance between accuracy and efficiency. Additionally, future extensions may incorporate temporal–frequency attention mechanisms. Recent studies have demonstrated that frequency-aware and cross-modal attention approaches, such as Fourier-based attention [29] and wavelet-based attention [30, 31], improved neural signal analysis in EEG domains by effectively modeling non-stationary neural signals [32] and enhancing nonlinear representation capacity [33, 34]. While the present study focused on spatial attention for sensor-efficient EEG channel reconstruction, integrating temporal–frequency attention modules represents a promising direction for further improving reconstruction fidelity and robustness.

## Conclusion

This study proposed VEEG-A-U-Net, a compact and effective framework for virtual EEG channel generation that bridges the gap between LCD and HCD configurations via a sensor-efficient, attention-guided design. Comprehensive evaluations on the SEED dataset showed that the proposed model achieved competitive reconstruction performance compared with traditional interpolation methods and several state-of-the-art deep learning approaches, as reflected by NMSE, SNR, and PCC. The ablation study further confirmed the contribution of attention gates, which enabled the network to dynamically focus on relevant spatiotemporal features, thereby enhancing reconstruction fidelity. Cross-dataset experiments were conducted using MI and MF EEG data to assess generalizability. The results confirmed the model’s robustness across varying EEG paradigms and recording conditions. In addition, a preliminary clinical EEG evaluation was conducted as a pilot feasibility study. However, it should be noted that this clinical evaluation involved only a single patient. While it provides initial evidence of reconstruction capability in a realistic clinical environment, future studies with larger and more diverse patient cohorts are required to avoid overgeneralization and fully validate the clinical robustness of the proposed framework. Notably, despite its markedly reduced model complexity and the absence of additional sophisticated modules, VEEG-A-U-Net achieved reconstruction performance comparable to current state-of-the-art methods. These findings indicate that, under equivalent reconstruction settings, the incorporation of attention gates into a U-Net architecture is sufficient to enable competitive EEG reconstruction within a lightweight framework, while also demonstrating practical feasibility for deployment in resource-constrained and edge computing environments.

## Supplementary Information

Below is the link to the electronic supplementary material.

Supplementary Material 1 (PDF 11.1 MB)

## Data Availability

The source code and model configurations of VEEG-A-U-Net are publicly available at https://github.com/IASlab526/VEEG-A-U-NetAdditional details are provided in the Supplementary Material (Supplementary.pdf).

## References

[1] S. J. Kim, *et al*., “Towards Domain-free Transformer for Generalized EEG Pre-training,” *IEEE transactions on neural systems and rehabilitation engineering*, vol. 32, pp. 482–492, Jan. 2024, doi: 10.1109/tnsre.2024.3355434 38236672 10.1109/TNSRE.2024.3355434

[2] R. T. Schirrmeister *et al*., “Deep learning with convolutional neural networks for EEG decoding and visualization,” *Human Brain Mapping*, vol. 38, no. 11, pp. 5391–5420, Aug. 2017, doi: 10.1002/hbm.23730 28782865 10.1002/hbm.23730 PMC5655781

[3] D. J. McFarland and J. R. Wolpaw, “EEG-based brain–computer interfaces,” *Current Opinion in Biomedical Engineering*, vol. 4, pp. 194–200, Dec. 2017, doi: 10.1016/j.cobme.2017.11.004 29527584 10.1016/j.cobme.2017.11.004 PMC5839510

[4] X. Chen, B. Zhao, Y. Wang, and X. Gao, “Combination of high-frequency SSVEP-based BCI and computer vision for controlling a robotic arm,” *Journal of Neural Engineering*, vol. 16, no. 2, p. 026012, Jan. 2019, doi: 10.1088/1741-2552/aaf594 10.1088/1741-2552/aaf594 30523962

[5] R. Abiri, *et al*., “A Usability Study of Low-Cost Wireless Brain-Computer Interface for Cursor Control Using Online Linear Model,” *IEEE Transactions on Human-Machine Systems*, vol. 50, no. 4, pp. 287–297, Aug. 2020, doi: 10.1109/thms.2020.2983848 33777542 10.1109/thms.2020.2983848 PMC7990128

[6] A. Corley and Y. Huang, “Deep EEG super-resolution: Upsampling EEG spatial resolution with generative adversarial networks”, *Proc. IEEE EMBS Int. Conf. Biomed. Health Inf.*, pp. 100–103, 2018, doi: 10.1109/BHI.2018.8333379

[7] A. C. K. Soong, *et al*., “Systematic comparisons of interpolation techniques in topographic brain mapping,” *Electroencephalography and Clinical Neurophysiology*, vol. 87, no. 4, pp. 185–195, Oct. 1993, doi: 10.1016/0013-4694(93)90018-q 7691549 10.1016/0013-4694(93)90018-q

[8] L. Shaw and A. Routray, “A New Framework to Infer Intra- and Inter-Brain Sparse Connectivity Estimation for EEG Source Information Flow,” in *IEEE Sensors Journal*, vol. 18, no. 24, pp. 10134–10144, 15 Dec.15, 2018, doi: 10.1109/JSEN.2018.2875377

[9] L. Dong *et al*., “Reference Electrode Standardization Interpolation Technique (RESIT): A Novel Interpolation Method for Scalp EEG,” *Brain Topography*, vol. 34, no. 4, pp. 403–414, May 2021, doi: 10.1007/s10548-021-00844-2 33950323 10.1007/s10548-021-00844-2 PMC8195908

[10] R. Liu, Z. Wang, J. Qiu, and X. Wang, “Assigning channel weights using an attention mechanism: an EEG interpolation algorithm,” *Frontiers in neuroscience*, vol. 17, Sep. 2023, doi: 10.3389/fnins.2023.1251677 10.3389/fnins.2023.1251677 PMC10552919 37811329

[11] M. Svantesson, *et al*., “Virtual EEG-electrodes: Convolutional neural networks as a method for upsampling or restoring channels,” *Journal of Neuroscience Methods*, vol. 355, p. 109126, May 2021, doi: 10.1016/j.jneumeth.2021.109126 33711358 10.1016/j.jneumeth.2021.109126

[12] L.-L. Li, *et al*., “EEG generation of virtual channels using an improved Wasserstein generative adversarial networks,” in *Proc. Int. Conf. Intell. Robot. Appl.* Cham, Switzerland: Springer, 2022, pp. 386–399. doi: 10.1007/978-3-031-13841-6_36

[13] Y. Tang, *et al*., “Deep EEG Superresolution via Correlating Brain Structural and Functional Connectivities,” in *IEEE Transactions on Cybernetics*, vol. 53, no. 7, pp. 4410–4422, July 2023, doi: 10.1109/TCYB.2022.3178370 35700255 10.1109/TCYB.2022.3178370

[14] H. Sun, C. Li, and H. Zhang, “Design of virtual BCI channels based on informer,” *Frontiers in human neuroscience*, vol. 17, Apr. 2023, doi: 10.3389/fnhum.2023.1150316 10.3389/fnhum.2023.1150316 PMC10165084 37169016

[15] G. Li et al., “A Super-Resolution Framework for Emotion Recognition Based on EEG Signals,” in *IEEE Sensors Journal*, vol. 24, no. 23, pp. 40137–40147, 1 Dec.1, 2024, doi: 10.1109/JSEN.2024.3484413

[16] W. L. Zheng and B. L. Lu, “Investigating Critical Frequency Bands and Channels for EEG-Based Emotion Recognition with Deep Neural Networks,” *IEEE Transactions on Autonomous Mental Development*, vol. 7, no. 3, pp. 162–175, Sep. 2015, doi: 10.1109/tamd.2015.2431497

[17] A. Delorme and S. Makeig, “EEGLAB: an open source toolbox for analysis of single-trial EEG dynamics including independent component analysis,” *Journal of Neuroscience Methods*, vol. 134, no. 1, pp. 9–21, Mar. 2004, doi: 10.1016/j.jneumeth.2003.10.009 15102499 10.1016/j.jneumeth.2003.10.009

[18] J. Kim, J. K. Lee, and K. M. Lee, “Accurate Image Super-Resolution Using Very Deep Convolutional Networks,” 2016 *IEEE Conference on Computer Vision and Pattern* Recognition *(CVPR)*, Jun. 2016, doi: 10.1109/cvpr.2016.182

[19] O. Oktay *et al*., “Attention U-Net: Learning Where to Look for the Pancreas,” arXiv.org, May 20, 2018. https://arxiv.org/abs/1804.03999v3

[20] C.-H. Chuang, *et al*., “IC-U-Net: A U-Net-based Denoising Autoencoder Using Mixtures of Independent Components for Automatic EEG Artifact Removal,” *NeuroImage*, vol. 263, p. 119586, Nov. 2022, doi: 10.1016/j.neuroimage.2022.119586 36031182 10.1016/j.neuroimage.2022.119586

[21] M. Kwon, *et al*., “Super-Resolution for Improving EEG Spatial Resolution using Deep Convolutional Neural Network—Feasibility Study,” *Sensors*, vol. 19, no. 23, p. 5317, Dec. 2019, doi: 10.3390/s19235317 10.3390/s19235317 PMC6928936 31816868

[22] S. Agarwal, *et al*., “EEG signal enhancement using cascaded S-Golay filter,” *Biomedical Signal Processing and Control*, vol. 36, pp. 194–204, Jul. 2017, doi: 10.1016/j.bspc.2017.04.004

[23] Y. Zhang, *et al.*, “MASER: Enhancing EEG Spatial Resolution With State Space Modeling,” in *IEEE Transactions on Neural Systems and Rehabilitation Engineering*, vol. 32, pp. 3858–3868, 2024, doi: doi: 10.1109/TNSRE.2024.3481886 39412979 10.1109/TNSRE.2024.3481886

[24] D. Li, *et al*., “ESTformer: Transformer utilising spatiotemporal dependencies for electroencephalogram super-resolution,” *Knowledge-Based Systems*, vol. 317, 113345, 2025, 10.1016/j.knosys.2025.113345

[25] L. Shaw and A. Routray, “Brain State Classification With Group l1 – norm Sparse PDC as Novel Features for EEG,” in *IEEE Sensors Journal*, vol. 21, no. 12, pp. 13506–13513, 15 June15, 2021, doi: 10.1109/JSEN.2021.3068125

[26] G. Schalk, *et al.*, “BCI2000: A General-Purpose Brain-Computer Interface (BCI) System,” *IEEE Transactions on Biomedical Engineering*, vol. 51, no. 6, pp. 1034–1043, Jun. 2004, doi: 10.1109/tbme.2004.827072 10.1109/TBME.2004.827072 15188875

[27] Adaptive EEG Channel Phase Synchronization. GitHub repository. [Online]. Available: https://github.com/IASlab526/Adaptive-EEG-Channel-Phase-Synchronization Accessed: Jan. 12, 2026.

[28] pytorch-OpCounter (THOP): Count the MACs / FLOPs of PyTorch models. GitHub repository. [Online]. Available: https://github.com/Lyken17/pytorch-OpCounter. Accessed: Jan. 12, 2026.

[29] H. Ke *et al*., “Unsupervised deep frequency-channel attention factorization to non-linear feature extraction: A case study of identification and functional connectivity interpretation of Parkinson’s disease,” *Expert Systems with Applications*, vol. 243, 122853, 2024, doi: 10.1016/j.eswa.2023.122853

[30] F. Wang *et al*., “Deep wavelet self-attention non-negative tensor factorization for non-linear analysis and classification of fMRI data,” *Applied Soft Computing*, vol. 182, 113522, 2025, doi: 10.1016/j.asoc.2025.113522

[31] F. Wang *et al*., “Deep wavelet temporal-frequency attention for nonlinear fmri factorization in asd. *Pattern Recognition*, vol. 165, 111543, 2025, doi: 10.1016/j.patcog.2025.111543

[32] F. Wang *et al*., “Fusion of generative adversarial networks and non-negative tensor decomposition for depression fMRI data analysis. *Information Processing & Management*, vol. 62, no. 2, 103961, 2025, doi: 10.1016/j.ipm.2024.103961

[33] H. Ke *et al*., “Deep Factor Learning for Accurate Brain Neuroimaging Data Analysis on Discrimination for Structural MRI and Functional MRI, ” in *IEEE/ACM Transactions on Computational Biology and Bioinformatics*, vol. 21, no. 4, pp. 582–595, July-Aug. 2024, doi: 10.1109/TCBB.2023.3252577 10.1109/TCBB.2023.3252577 37028037

[34] H. Ke *et al*., “ADHD identification and its interpretation of functional connectivity using deep self-attention factorization,” *Knowledge-Based Systems*, vol. 250, 109082, 2022, doi: 10.1016/j.knosys.2022.109082

