# Supplementary material for: Attention-Enhanced U-Net for Sensor-Efficient High-Density EEG Reconstruction in Wearable Brain Monitoring Systems
Source: J Med Syst. 2026 Apr 6;50(1):46. doi: 10.1007/s10916-026-02374-5 (PMC13050758; doi:10.1007/s10916-026-02374-5)
Supplement: Supplementary file 1 — Supplementary Material 1 (PDF 11.1 MB) [file 10916_2026_2374_MOESM1_ESM.pdf]

Supplementary

Visualization and Subject-Level Analysis of Attention Weight Distributions

Figures S1–S16 present visualization analyses of the attention weight distributions learned by the proposed VEEG-A-U-Net. Specifically, scalp heatmaps of attention weights over the original 60 EEG channels are extracted from the outputs of the decoder attention gates.

The results include both the average attention distribution across all subjects (AllSub) and individual subject-level maps (Sub01–Sub15), enabling detailed inspection of spatial consistency, inter-subject variability, and reproducibility of the learned attention patterns.

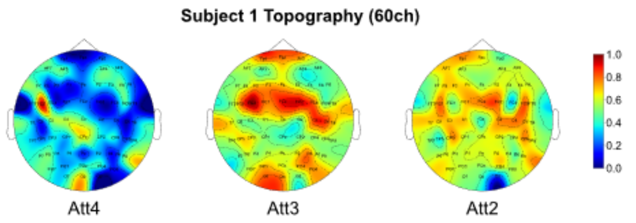

Figure S1 Subject 1 Topography

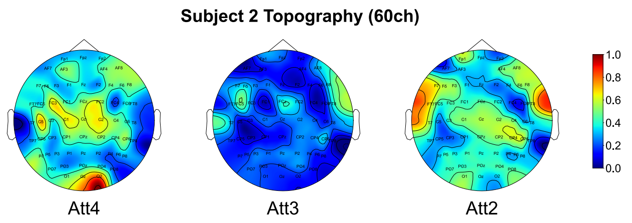

Figure S2 Subject 2 Topography

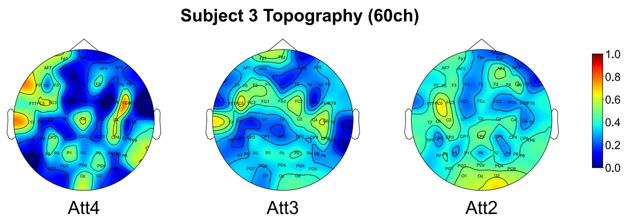

Figure S3 Subject 3 Topography

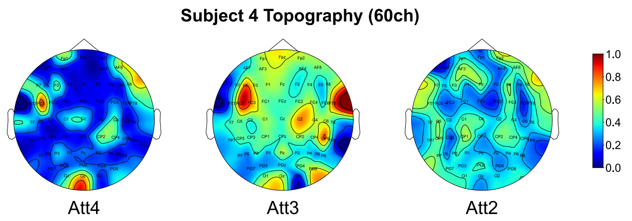

Figure S4 Subject 4 Topography

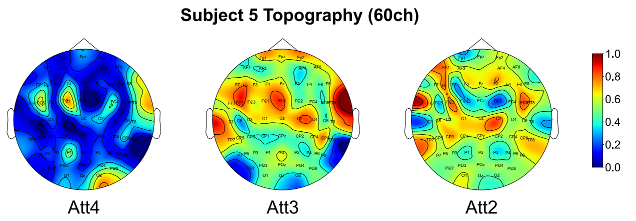

Figure S5 Subject 5 Topography

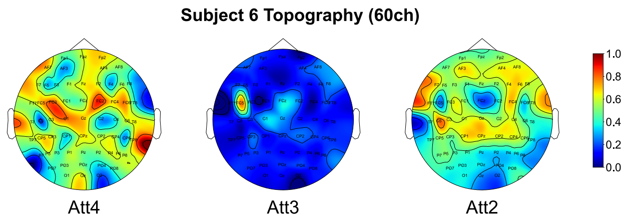

Figure S6 Subject 6 Topography

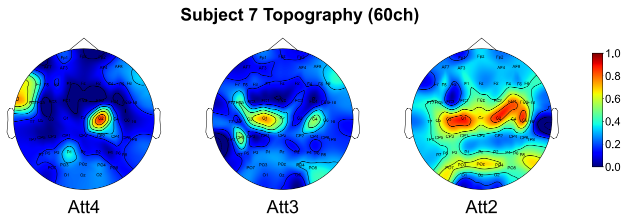

Figure S7 Subject 7 Topography

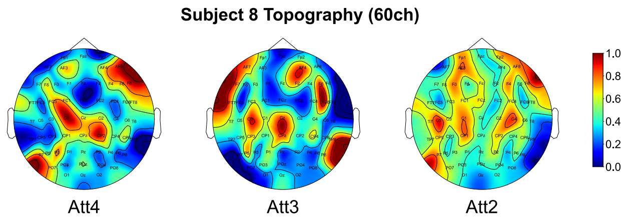

Figure S8 Subject 8 Topography

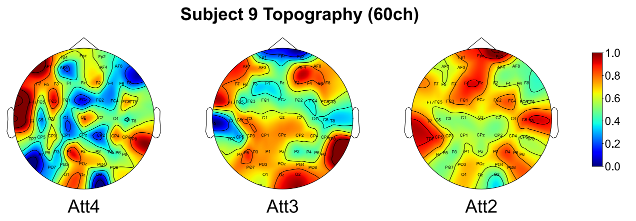

Figure S9 Subject 9 Topography

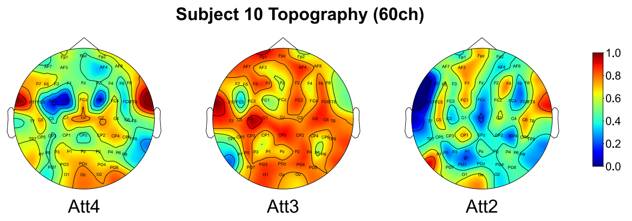

Figure S10 Subject 10 Topography

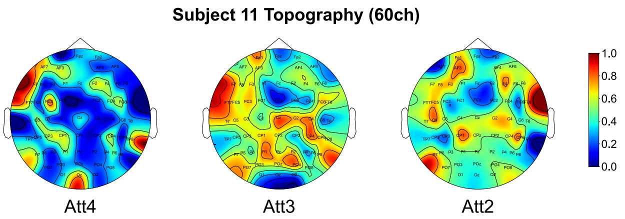

Figure S11 Subject 11 Topography

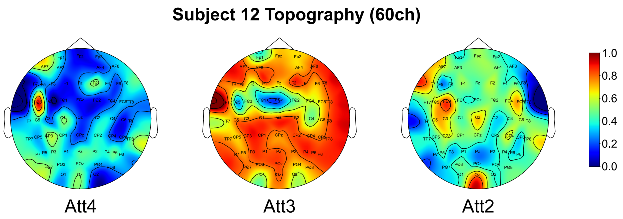

Figure S12 Subject 12 Topography

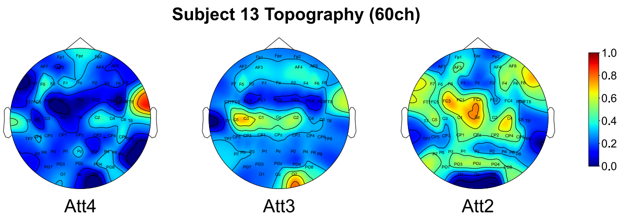

Figure S13 Subject 13 Topography

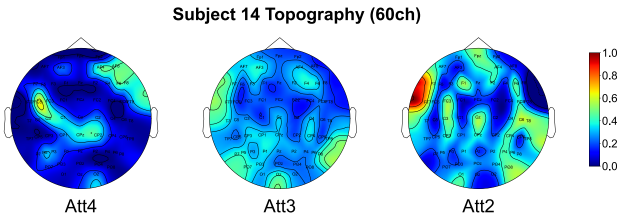

Figure S14 Subject 14 Topography

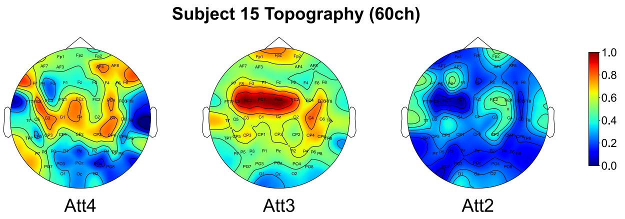

Figure S15 Subject 15 Topography

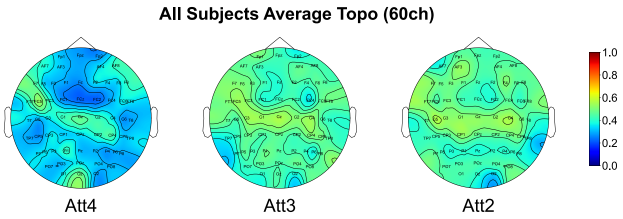

Figure S16 All Subjects Average Topography
